# Living in Roma Settlements in Greece: Self-Perceived Health Status, Chronic Diseases and Associated Social Determinants of Health

**DOI:** 10.3390/ijerph18168403

**Published:** 2021-08-09

**Authors:** Ioanna Petraki, Natasa Kalpourtzi, Agapios Terzidis, Magda Gavana, Apostolos Vantarakis, Georgios Rachiotis, Argiro Karakosta, Vana Sypsa, Giota Touloumi, Hprolipsis Study Group

**Affiliations:** 1 Department of Hygiene, Epidemiology, and Medical Statistics, Medical School, National and Kapodistrian University of Athens, 75, Mikras Asias Str., Goudi, 11527 Athens, Greece; akalpourtzi@med.uoa.gr (N.K.); akarakosta@med.uoa.gr (A.K.); vsipsa@med.uoa.gr (V.S.); gtouloum@med.uoa.gr (G.T.); 2 International Medicine-Health Crisis Management, School of Medicine, National and Kapodistrian University of Athens, 1, Dilou & Mikras Asias Str., Goudi, 11527 Athens, Greece; agisterzidis@gmail.com; 3 Department of Primary Health Care, General Practice, and Health Services Research, Medical School of Aristotle University, 54124 Thessaloniki, Greece; magda.gavana@gmail.com; 4 Environmental Microbiology Unit of Public Health, Medical School, University of Patras, Rio Achaia, 26504 Patra, Greece; avantar@med.upatras.gr; 5 Department of Hygiene and Epidemiology, Medical Faculty, University of Thessaly, 3, Panepistimiou Str., Viopolis, 41500 Larisa, Greece; grachiotis@gmail.com

**Keywords:** Roma settlements, Greece, self-perceived health, chronic diseases, social determinants of health

## Abstract

We aimed to assess the self-perceived health status and the presence of chronic diseases of adult Roma living in settlements in Greece, and to explore associated social determinants of health. Data were derived from the Hprolipsis Health Survey. Multivariable regression models were applied. In total, 534 adults, 287 women, and 247 men were recruited from twelve Roma settlements in four prefectures. Although 62% of the participants perceived their health status as good/very good, about half of them had been diagnosed with at least one chronic disease. Several structural and intermediary social determinants of health were found to be significantly associated with the health outcomes; prefecture, settlement type, sex, age group, living with a partner, presence of depression symptoms, food insecurity, and alcohol consumption were associated with self-perceived health status; settlement type, sex, age group, presence of anxiety symptoms, food insecurity and number of persons living in the house with the presence of a chronic disease. This is one of the few studies assessing the self-perceived health status and presence of chronic diseases in Roma settlements in Greece and investigating the associated social determinants of health in the world. Community-based participatory action research and health literacy programs are needed to mitigate health inequalities in Roma settlements.

## 1. Introduction

Who are the Roma? What is the best term to describe them? What is their number around the world? All the above are subject of continuous debate [1,2]. However, by the umbrella term “Roma”, we usually refer to the largest ethnic minority in Europe of 10 to 12 million people who originated from India and have faced centuries of discrimination and social exclusion. Due to this exclusion, the majority of Roma live in overwhelmingly poor conditions on the margins of society [3,4].

Roma may have approached the Byzantine Empire around the 10th century [5], while by 1435 they had spread across most European cities [6]. The first recorded sale and purchase of Roma slaves took place in 1385 in Moldavia [7]. From the 16th century onwards, increasingly extreme legislation enacted in many European countries led to expulsions, deportations, and forced assimilation, culminating in the organized killing of the Romani people [8]. The second wave of immigration began in the middle of the 19th century [9]. During World War II, the Roma were defined as “problem”, “anti-social”, and “racially inferior”, and were arrested and killed in German-occupied territories [10]. In the 1950s and 1960s, the first Romani organizations were established [1]. With the collapse of communism in Europe, as well as the abolition of the restrictions on the movement of people from Central and Eastern European countries that took place after 1989, the third European period of Roma dispersion begun [11]. 

Since the 1990s, the Roma have been the target of Europe-wide development programs and discourses. However, the living conditions of the poorest among the Roma worsened, while racist violence and biopolitical methods of eugenics—for instance, the sterilization of Romani women without their consent—increased [12,13]. For the past 30 years, the European Union (EU) Framework for National Roma Integration Strategies has been one of the main evolving policy instruments targeting Roma inclusion, promoting access to education, employment, health, and housing. These policies also led to the development of anti-discrimination and anti-gypsyism campaigns [14]. Even though there has been some progress, long-term results remain limited [15].

Today, the Roma experience more severe poverty and social exclusion than almost all the other minority groups in the countries they live, which is compounded and intensified by deep-seated prejudice, racism, and discrimination [16]. This multidimensional nature of the exclusion and marginalization that the Romani people have faced for centuries is not only due to a lack of financial resources, unemployment, sub-standard housing conditions, or limited access to health and social services, but is rather due to the combination of all these factors. These factors are both the outcomes of a history of exclusion and the determinants of future exclusion—reinforcing the vicious circle of poverty [17].

The Roma have poorer health status, including higher rates of both communicable (e.g., hepatitis A and B) and non-communicable (e.g., cardiovascular diseases, diabetes mellitus) diseases compared to non-Roma [18,19,20,21,22]. The health disparities experienced by the Romani people are most commonly attributed to the social determinants of health [18,19]. More specifically, the “causes of the causes” of these health disparities are the conditions in which people are born, grow, live, work, and age as well as the inequities in power, money, and resources that give rise to them [23].

The Commission on Social Determinants of Health which was set up by the World Health Organization to work to the heart of this complexity has proposed a single conceptual framework for action on the social determinants of health. The core components of the Commission on Social Determinants of Health conceptual framework, include: (a) socioeconomic and political context (governance, macroeconomic, social and public policies, culture, and societal values); (b) structural determinants of health inequities (socioeconomic position, social class, gender, ethnicity, education, occupation, and income); and (c) intermediary determinants of health (material and psychosocial circumstances, behavioral, and/or biological factors) [24].

It has become clear that there is a need to establish national and global health equity surveillance systems with a routine collection of data on social determinants of health [25]. Despite this, reliable research evidence on Roma health is limited, as most available data are based on anecdotal evidence, poorly-designed descriptive, localized, and community-level epidemiological studies with inadequate sample sizes and of poor reliability [20,21,22]. In addition, the topics that have received attention among the researchers imply a focus on concepts of contagion or social Darwinism, indicating a greater concern with the health needs of the majority populations rather than the ones of Roma [26]. Adding to that, a number of studies highlight the need for further studies on non-communicable diseases and the Roma [20,27,28], taking into account the challenges and dilemmas researchers working with such stigmatized populations may encounter (e.g., Roma conceptualization, sampling limitations, ethnic identity, etc.) [29,30].

In Greece, the Roma became beneficiaries of basic and fundamental social rights only in 1955, when they were recognized as citizens of the country [31]. In the 1980s, the first Roma integration programs appeared, but social inclusion did not become an objective of government policy until the mid-1990s [32]. Contrary to most of the EU countries, the Roma are not officially recognized in Greece as a national or linguistic minority, and the available data on their health is limited and fragmented, especially regarding non-communicable diseases. To our knowledge, six cross-sectional surveys that have provided data on Roma health from various prefectures of Greece (at least four different ones), have been conducted till today [33,34,35,36,37,38]. Interestingly, only the results of one of these [38] have been published in a peer-reviewed journal and it is the only one that explores the effect of determinants on the health of the Romani people in Greece [38]. In particular, this study assessed the health-related quality of life of Greek Roma living in Roma settlements and explored its association with socio-economic factors, housing conditions, and self-perceived health status [38]. However, the study sample included only individuals who visited the Social Medical Centers, and thus, it was far from being representative of the Roma population living in Greece.

Our objective was to assess the self-perceived health status and the prevalence of self-reported chronic diseases among adult Roma who live in different types of Roma settlements in Greece and to explore their association with social determinants of health. Our study was designed to collect several data on structural, such as gender, education, and income, and intermediary, such as behavioral risk factors and material circumstances, social determinants of health, based on the Commission on Social Determinants of Health conceptual framework [24]. Also, we aimed to recruit, as much as possible, a representative sample of Roma from all types of settlements (houses, houses and shacks (mixed), and shacks) [39], addressing, thus, one of the major gaps in the existing limited literature.

## 2. Materials and Methods

Data were derived from the Hprolipsis Health Survey. The study design has been previously reported [39]. Hprolipsis consists of three cross-sectional epidemiological surveys of three adult (≥18 years) populations: (1) the general population, (2) Roma living in Roma settlements (Roma who live in houses dispersed among the majority population were part of the general population survey and thus were excluded from the Roma component), and (3) migrants. The data collection for the Roma component was initiated in December 2014 and completed in January 2016. The Roma Health Survey was based on non-probability multistage quota sampling and the target sample size was set to approximately 500 adults.

Data were collected from four prefectures in Greece. Three of the prefectures contained larger populations of Romani people: Peloponnese (Western Greece), Thessaly (Central Greece), and Central Macedonia (Northern Greece) [37]. One prefecture contained the largest urban center in Greece (Attica). In each selected prefecture, Roma settlements representative of three settlement types were chosen: houses, houses and shacks (mixed), and shacks. In collaboration with Roma cultural mediators, all adult Roma living in these specific settlements were invited to participate in the study (external identification of Roma participants), while maintaining the desired and predetermined distribution by gender and age.

### 2.1. Questionnaire

A questionnaire based on guidelines for health examination surveys [40] was developed [39,41]. The Hprolipsis Health Survey questionnaire for the Roma component was initially developed and pretested with four Roma volunteers following established protocols. Standardized equipment and instruments were used, when possible. A description of the equipment and instrument used as well as the source of the sub-questionnaires have been described elsewhere [39,41].

Socio-demographic characteristics, behavioral risk factors and material circumstances indicators were collected. Participants were also asked about their self-perceived health status (very good, good, fair, bad, very bad) and presence of a diagnosed chronic health condition lasting more than six months (high cholesterol, diabetes mellitus, high blood pressure, heart disease, respiratory system disease, tuberculosis, cancer, anemia, depression, any other chronic disease/specify).

Participants were asked about their drinking water supply (house tap water/public tap water/other), electricity supply (public power corporation/generator/no electricity), heating (yes/no), and toilet inside the house (yes/no). Regarding main health-related behavioral risk factors, participants were asked about alcohol consumption (yes/no), and if yes, how much during the last week (using a portion measurement), and how many days they consumed alcohol. The smoking status (smoker/past smoker/never smoker) and the number of cigarettes consumed if a smoker or past smoker (how many cigarettes for how many years) was also determined through the survey.

The 4-item Patient Health Questionnaire (PHQ-4) that consists of a 2-item depression scale (PHQ-2) and 2-item anxiety scale (GAD-2) [42] was administered to screen for the presence of anxiety and depression symptoms (Table A1). A score ≥ 3 in PHQ-2 and GAD-2 indicates the presence of anxiety and depression symptoms, respectively. A 3-item, each scoring 0–2, household food insecurity questionnaire was also administered (Table A2) [43], and based on the total calculated score participants were categorized as (a) not having (score = 0), (b) rare or sometimes having (score = 1–3), and (c) often having food insecurity (score = 4–6) [44].

Based on participants’ replies, three new variables were created. “>1 chronic disease” variable, indicating the presence of more than one chronic condition per participant. “Basic household facilities” variable, calculating the number of the four available household facilities (water, electricity, heating, and toilet inside the house) inside each house (all 4/2–3/0–1); the availability of these facilities was defined as “House tap water” in the water supply, “Public Power Corporation” or “Generator” in the electricity supply and “Yes” for the presence of heating and a toilet inside the house. Last, the “smoking pack-years” variable, assuming that a package of cigarettes has 20 cigarettes.

### 2.2. Statistical Analysis

The means and standard deviation (SD) or medians and interquartile range (IQR) were used for describing continuous variables and percentages were used for categorical variables, as appropriate. The frequencies of socio-demographic characteristics and behavioral risk factors were calculated by settlement type, whereas the frequencies of self-perceived health status, the presence of chronic diseases, and anxiety and depression symptoms variables were calculated by gender. Differences by settlement type or gender were tested using the chi-square test, except for one case where Fisher’s Exact Test was used, as there was only one positive answer.

Univariable and multivariable logistic regression models were fitted to investigate the association of selected variables with the self-perceived health status analyzed as a dichotomous variable (very good and good vs. fair, bad, very bad self-perceived health status) and with the presence of at least one chronic disease (no/yes). For these analyses, potential risk factors were categorized as: age “18–29/30–45/46+” years; family status “living with a partner/not living with a partner”; the number of children “0–3/4+” (this categorization was chosen as in Greece, families with 4 children or more receive specific benefits); educational status “have been to school/have never been to school”; job status “working/not working”; food insecurity “never/rare, sometimes, often”; basic household facilities “all 4/2–3/0–1”; smoking “ever smoker/never smoker”, and income as “have income/not have income”.

All analyses were performed using the Stata software version 13.0 with statistical significance set at *p* < 0.05.

### 2.3. Ethics

Hprolipsis was approved by the Bioethics Committee of the National and Kapodistrian University of Athens (date: 4 March 2015, protocol number: 6141). All participants were given enough time to carefully read the participant information and consent forms and to ask relevant questions before they signed. Study participant information and consent forms were linguistically simplified for Roma participants and were explained to them if illiterate. Cultural mediators were also available. The questionnaire was anonymized. Participants’ personal data and their link to individual code were safely stored in a separate file from the main database.

## 3. Results

### 3.1. Socio-Demographic Characteristics

A total of 534 adults (287 women and 247 men) living in three settlement types in the four study prefectures were recruited. The socio-demographic characteristics, behavioral risk factors, and material circumstances of the study population overall and by settlement type are presented in Table 1. Most of the study participants were living with a partner (80%) and half of them were between 25–48 years old (IQR). The median age across all participants was 35 years, with the median age of the women being slightly older at 38 than the men at 31 years old. Almost half (46%) had four or more children.

Overall, the participants experienced limited schooling and employment opportunities. In line with lack of schooling and employment opportunities, participants reported difficulties providing for their needs and obtaining health insurance. More than half of the participants (50.7%) had never been to school and only 9% had attended at least some years of secondary school. Less than 30% were employed, and an even smaller percentage (16%) earned more than the lowest basic salary in Greece (586 Euros gross). Almost a quarter (22%) reported never having worked for payment. Only about 7% reported that they could cover their needs, less than half (48.5%) had health insurance, and almost one in four (24.5%) lived in households in which at least one person regularly went to bed hungry in the preceding month. Compared to men, women were less likely to have attended school or to have a job (approximately 65% of women had never been to school and 88% of them did not have a job, opposed to 33% and 48% of men, respectively). More than 90% had been staying for more than 6 years in the same area. Those who reported moving once every 6 or 12 months (12.6%) did so for job reasons (95.7%) rather than lifestyle reasons (4.3%). Almost half of the participants (47.2%) reported that approximately four or more persons sleep in the same bedroom. Regarding basic household facilities (water, electricity, heating, and toilet inside the house), only 4% of the participants had all four of them available in their house, versus approximately 77% and 18% that had 2–3 and 0–1, respectively.

Participants from settlements with shacks were younger, more likely to live with a partner, and had more children compared to participants from settlements with houses and mixed settlements. Also, they were more likely to sleep with four or more people per bedroom, they had the least basic household facilities, they had higher food insecurity scores, and they changed more often the place of residence compared to the others. On the other hand, participants from settlements with houses seemed to have the highest number of people who did not have an income, and participants from mixed settlements had the lowest number of people with health insurance and the highest number of glasses of alcohol consumption per last week.

### 3.2. Self-Perceived Health Status, Chronic Disease and Mental Health Symptoms

Participants’ self-perceived health status, the presence of diagnosed chronic diseases, and the presence of anxiety and depression symptoms are presented in Table 2 by gender. The answers “Don’t Know/Not-Available”, ranging from 6% to 10%, were omitted. Approximately 62% responded that they had good/very good health, 26% that they had fair, and 12% that they had bad/very bad. Women reported worse self-perceived health compared to men. Only a quarter of women aged 46 years and over (26%) reported good/very good health compared to half (55%) of the men of the same age.

Participants from settlements with shacks had the highest percentage of good/very good self-perceived health status responses (66.4%) compared to those from settlements with houses (56.4%) and mixed settlements (63.2%). Also, a quarter of those who had been to school and half of those who had not been to school reported fair/bad/very bad health.

Approximately half of the participants in total and one in every three of those with good/very good self-perceived health reported having at least one chronic disease. About 37% of those who had, and 62% of those who had not been to school responded that they have at least one chronic disease. Participants from settlements with shacks had the lowest percentage of people with a chronic disease, in comparison with participants from settlements with houses and mixed settlements (44.9%, 57.4%, and 45.2, respectively). Approximately 29% of people aged 18–29, 46.6% aged 30–45, and 76.3% aged 46 years and over responded they had a chronic disease.

The percentage of having at least one diagnosed chronic disease was higher in women compared to men. Additionally, among women, the percentages of those with symptoms of anxiety and of depression were very high, significantly higher than those among men.

### 3.3. Factors Associated with Good/Very Good Self-Perceived Health Status; Results from Multivariable Analysis

All socio-demographic factors presented in Table 1, as well as symptoms of anxiety or depression, were investigated for their potential association with self-perceived health status. Results from the final multivariable logistic regression model that included all statistically significant factors among those investigated are presented in Table 3. Overall, participants from Thessaly were almost four times and participants from Peloponnese almost two times more likely to report good/very good self-perceived health status compared to those from Attica, whereas there was no statistically significant difference in the odds of reporting good/very good self-perceived health status between those from Attica and from Central Macedonia. Participants from settlements with houses and, to some degree from mixed settlements, were less likely to report good/very good self-perceived health status as compared to those living in settlements with shacks (Adjusted OR (95% CI): 0.46 (0.25 to 0.86) and 0.76 (0.42 to 1.36), respectively).

Women, those with symptoms of depression, and those who experienced some food insecurity were less likely to report good or very good self-perceived health status. On the other hand, those living with a partner were more likely to report good or very good self-perceived health status. Older participants were less likely to report good or very good health status. Compared to the youngest age group (18–29 years), participants older than 45 years had a 90% reduced odds of reporting good or very good health. Those between the ages of 30–40 years had 54% reduced odds of reporting good or very good health compared to the youngest participants. A U shape association between the number of alcohol drinks consumption and the odds of reporting good or very good self-perceived health status was found, with those consuming 1–7 drinks per week having increased odds by 124% and those consuming 8 or more drinks per week having reduced odds by 10% to report good or very good self-perceived health status as compared to those not consuming alcohol, although the latter difference is not statistically significant.

### 3.4. Factors Associated with the Presence of a Chronic Disease; Results from Multivariable Analysis

Results from the adjusted analysis investigating the same variables as in the previous analysis are presented in Table 4. Those living in settlements with houses were 70% more likely to report at least one chronic disease compared to those living in settlements with shacks. Women, those with symptoms of anxiety, and those who experience some type of food insecurity have higher odds of reporting at least one chronic disease. As expected, older age was associated with higher odds of having at least one chronic disease with those aged >45 years old having more than 8 times, and those aged 30–45 years almost two times higher odds of having at least one chronic disease as compared to those aged 18–29 years. Interestingly, those living with up to 5 persons in the same house have 62% higher odds of reporting at least one chronic disease compared to those who are living with 6 or more persons in the same house.

## 4. Discussion

This is one of the few studies assessing the self-perceived health status and presence of chronic diseases in Roma living in different types of settlements in Greece and investigating the associated social determinants of health in the world. Our findings showed that, although approximately 62% of the participants perceived their health status as good or very good, about half of them had been diagnosed with at least one chronic disease, with women being in a worse situation than men.

The percentage of participants reporting perceived health status as good or very good is lower than that in the general population of Greece and the average of the Organization for Economic Co-operation and Development (OECD) 34 member countries in the same year (2015) (62%, 74%, and 68%, respectively) [45]. Similarly, the percentage of those having been diagnosed with at least one chronic disease is almost two times higher than that in the general population in Greece, and almost 13% higher than that in the EU-28 population [46]. Our finding of about half of the study participants with at least one chronic disease is similar to that found in the national surveys of the Ministry of Labor and Social Insurance (MLSI) [35] and in the survey of the Ministry of Employment and Social Protection (MESP) [37], but dissimilar to those found in the surveys of the Fundación Secretariado Gitano (FSG) [36] and the European Union Agency for Fundamental Rights (FRA) EUMIDIS II [47], in which the corresponding percentage was much lower (15.8% and 14%, accordingly).

Although the combined results of the FSG project [36], and the FRA pilot survey [48], and our study indicate an increasing trend in Roma reporting poor self-perceived health (8.3% in 2008, 11% in 2011, and 12.2% in 2016) the FRA EUMIDIS II survey [47] reported a lower estimated percentage for 2016 (7%). However, these results should be interpreted cautiously due to differences in the applied methodologies.

The high percentage of participants reporting they did not know if they had been diagnosed with a chronic disease in our study could be attributed to Roma’s low health literacy, and to the consequent lack of prevention culture [49,50]. Paradoxically, more participants from settlements with shacks in comparison to those from settlements with houses and mixed settlements, reported their self-perceived health as good or very good, even after adjusting for possible differences in sociodemographic characteristics; approximately one in every three participants from settlements with shacks who had a chronic condition reported good or very good self-perceived health status. A similar phenomenon was noted in the FSG [36] study, where Greek Roma participants, despite declaring a state of health in line with the European Roma average, seemed to have a higher incidence of disability or chronic disease.

The “positive” self-perception of general health among Roma is not a new finding, as, for example, the self-reflection of their health status, might be affected by lower access to health services, prejudices, and low health awareness [51]. Also, it might be the effect of the strong social ties of Roma with their core and extended family members, which, based on Antonovsky’s salutogenic model [52], seem to function as a resource for general resistance to stress and difficulties in life [21]. Another possible explanation is that the real health problems of Roma might only be perceived once they reach acute forms or require in-patient visits [51]. In any case, the above are just possible explanations for these phenomena, and therefore, future relevant studies should consider incorporating anthropological perspectives and tools that will allow to better interpret such “puzzles”.

To our knowledge, apart from the Pappa et al. study [38], there are no other studies investigating social determinants of health associated with the self-perceived health status and the presence of chronic diseases in Roma living in Greece, with which to compare our findings. However, several comments can be made. Our findings regarding the settlement type patterns (i.e., better self-perceived health status in those living in worse conditions), contrast with those of a study conducted in England [22]. Nevertheless, it is not possible from our cross-sectional data to determine whether the accommodation has an effect on health or vice versa (inverse causality). As expected, the percentage of bad or very bad self-perceived health status and of the presence of chronic diseases rises with age. However, it is important to take into consideration that the increase of reported health problems among older Roma is much steeper, reaching 70% for those 65 years old, compared with the corresponding 56% among the non-Roma [51].

The findings of the worse self-perception of health status and the higher percentages of chronic diseases, anxiety, and depression symptoms of Roma women in comparison to Roma men is in accordance with several similar Roma studies conducted in Greece [36,37,38,47] and other European countries [53,54]. It is also noteworthy that the percentage of those with depression or anxiety symptoms, which were also identified as risk factors, were almost two times higher among Roma participants than among the general population of the Hprolipsis study (36.7% vs. 14.2%, and 41.2% vs. 23.0%, respectively). (G. Touloumi, 2020, personal communication). Therefore, public health policymakers and practitioners ought to be aware of the common basic principle of gender dimension on Roma inclusion [55] and pay particular attention to the interplay of structural determinants of health, such as ethnicity, class, and gender-based discrimination, which is often overlooked and/or misunderstood [56].

Concerning other structural determinants of health, educational level, which is considered to be one of the most important determinants [57], was identified as a significant determinant in the Pappa et al. study [38], but this was not the case in our study. However, based on other findings [58], higher educational attainment is associated with better self-perceived health. Therefore, this “paradox” of our study could be attributed to the fact that only 9% of our participants had attained a secondary class and this is probably why such a difference could not be identified. A similar explanation could be valid for the family income which, likewise, was not found to be associated with self-perceived health in our study; income might not play an important role when comparing among Roma living in their communities as, in reality, the majority of them are very poor. This finding is in accordance with the one provided by a Slovakian study [59], but dissimilar to the one obtained by Masseria et al. [60].

Regarding intermediary social determinants of health, similar to our study, not having a partner was also found to be a risk factor of bad or very bad self-perceived health at the Pappa et al. survey [38], whereas the number of people living in the same house did not seem to be a significant determinant. However, the protective effect of having a partner in these two studies seems to contradict that of a Spanish study that showed that having a marital status other than single increases the probability of perceiving very poor health [54]. Last, food insecurity seems to be once more an intermediary social determinant of the health of particular concern among Roma living in settlements in Greece [61].

### 4.1. Strengths and Limitations

The main strength of our study was the use of a unified methodology and standardized instruments across all selected areas. The study was implemented according to the educational program “Young Roma Health Mediators” guidelines and, as such, had wide acceptance of the Roma communities, establishing trust relationships [62]. Our study is also one of the few studies providing sound quantitative evidence on the poor self-perceived health status and on chronic diseases self-reported prevalence and associated risk factors of Roma residing in Roma settlements in Greece, as most previous studies focused on infectious diseases. The use of health outcome variables enabled comparison with results from different research settings and provided a baseline for future comparisons, with an additional focus on social determinants in public health research for Roma [25].

Our study is also subject to some limitations. Our sample, in the absence of census data for Roma in Greece, was not probabilistic but was based on quota sampling. Nevertheless, the effort made to collect and synthesize information on different types of Roma settlements, the multistage sampling method used and the collaboration with experienced researchers, Roma mediators, and members from the Roma communities we visited suggesting that our findings are likely to be representative of Roma living in Roma settlements in Greece. Another limitation was that indicators used here (e.g., self-perception of their health status, presence of chronic disease, etc.), although standardized for the general population, could be very misleading when applied to Roma surveys, a population of low educational level and with differences in health perceptions. Moreover, collecting accurate information for some specific variables, such as the family income, was not feasible due to the sensitivity of these questions, which would have impacted our collaboration with the population. Further, it is never possible, with a cross-sectional study, to establish causality. Lastly, our study was questionnaire-based and consequently, information bias may have occurred.

### 4.2. Conclusions

Our study provided further evidence for the worse health condition of Roma residing in Roma settlement in Greece compared to the general population. Several social determinants of health, such as gender, settlement type, food insecurity, and age, were found to be independently associated with low self-perceived health status and the presence of chronic disease. As there is a lack of informative and comparable relevant indicators among different studies, greater efforts should be made to combine these indicators with solid and reliable medical information [51]. A salutogenic approach and the use of a sense of coherence scale could be combined as a valid, reliable, and cross-culturally applicable instrument for measuring health, which will allow a better sensible interpretation of such data [63]. Additional community-based participatory action research that will shed light on Roma’s health risk factors, as well as health literacy programs based on best practices are needed to mitigate health inequalities and social exclusion in Roma communities. Lastly, our study has policy implications related to the urgent need for improvement of the food insecurity conditions in Roma communities in Greece.

## 5. Highlights

- Twelve Roma settlements representative of three different settlement types in Greece (houses, mixed, and shacks).
- Unified methodology and standardized instruments across all selected areas.
- Sound quantitative evidence on the worse health condition of Roma residing in settlements in Greece compared to the general population.
- Several social determinants of health, including gender, food insecurity, and settlement type, were associated with increased odds of poor self-perceived health and with the presence of chronic diseases.

## Figures and Tables

**Table 1:** Socio-demographic characteristics and behavioral risk factors of Roma living in Roma settlements in Greece by settlement type (houses, mixed, and shacks).

	N (%)				*p*-Value
	Settlement Type			Total	
	Houses	Mixed	Shacks		
**Number of participants**	165 (30.9)	232 (43.4)	137 (25.7)	534	
**Prefecture**					<0.001
Attica	45 (27.3)	43 (18.5)	46 (33.6)	134 (25.1)	
Cent. Macedonia	36 (21.8)	42 (18.1)	41 (29.9)	119 (22.3)	
Peloponnese	30 (18.2)	93 (40.1)	20 (14.6)	143 (26.8)	
Thessaly	54 (32.7)	54 (23.3)	30 (21.9)	138 (25.8)	
**Gender**					0.515
women	94 (57.0)	124 (53.4)	69 (50.4)	287 (53.7)	
men	71 (43.0)	108 (46.6)	68 (49.6)	247 (46.3)	
**Age, years**					0.014
18–29	60 (36.4)	75 (32.3)	63 (46.0)	198 (37.1)	
30–45	60 (36.4)	67 (28.9)	169 (31.6)	169 (31.6)	
46–69	39 (23.6)	79 (34.1)	149 (27.9)	149 (27.9)	
70+	6 (3.6)	11 (4.7)	18 (3.4)	18 (3.4)	
**Family status**					<0.001
single	33 (20.1)	19 (8.2)	10 (7.3)	62 (11.6)	
married	70 (42.7)	93 (40.1)	47 (34.3)	210 (39.4)	
widowed/divorced	16 (9.8)	28 (12.1)	5 (3.6)	49 (9.2)	
“stolen”	45 (27.4)	92 (39.7)	75 (54.7)	212 (39.8)	
**Number of children**					0.004
0	30 (18.3)	19 (8.2)	10 (7.3)	59 (11.1)	
1–3	70 (42.7)	105 (45.3)	54 (39.4)	229 (43.0)	
4+	64 (39.0)	108 (46.6)	73 (53.3)	245 (46.0)	
**Educational level**					<0.001
never been to school	57 (34.5)	128 (55.4)	85 (62.0)	270 (50.7)	
up to 6 years (primary)	79 (47.9)	92 (39.8)	44 (32.1)	215 (40.3)	
more than 6 years (secondary)	29 (17.6)	11 (4.8)	8 (5.8)	48 (9.0)	
**Job status**					0.042
employed (permanently or temporarily)	40 (24.2)	65 (28.0)	48 (35.0)	153 (28.7)	
retired	9 (5.5)	8 (3.4)	1 (0.7)	18 (3.4)	
household, student, supporting family business (without salary)	54 (32.7)	64 (27.6)	48 (35.0)	166 (31.1)	
unemployed	62 (37.6)	95 (40.9)	40 (29.2)	197 (36.9)	
**Family income compared to lowest basic salary in Greece (586 € gross)**					0.004
higher/almost the same	37 (22.6)	27 (11.7)	20 (14.6)	84 (15.8)	
lower	78 (47.6)	137 (59.6)	90 (65.7)	305 (57.4)	
no income	49 (29.9)	66 (28.7)	27 (19.7)	142 (26.7)	
**Needs covered based on income**					0.001
no	144 (87.3)	219 (94.4)	134 (97.8)	497 (93.1)	
yes	21 (12.7)	13 (5.6)	3 (2.2)	37 (6.9)	
**Health insurance**					
no	63 (38.9)	137 (59.3)	73 (53.3)	273 (51.5)	
yes	99 (61.1)	94 (40.7)	64 (46.7)	257 (48.5)	
**Food insecurity (score)**					0.131
never (0)	62 (38.0)	91 (39.6)	37 (27.0)	190 (35.8)	
rare/Sometimes (1–3)	58 (35.6)	85 (37.0)	56 (40.9)	199 (37.5)	
often (4–6)	43 (26.4)	54 (23.5)	44 (32.1)	146 (26.6)	
**Years living in this area**					0.016
up to 5	14 (8.5)	13 (6.0)	20 (15.0)	47 (9.1)	
6+	151 (91.5)	204 (94.0)	114 (85.0)	468 (90.9)	
**Change place of residence**					0.012
no	142 (87.1)	212 (91.4)	109 (80.7)	463 (87.4)	
yes, once per 6 or 12 months	21 (12.9)	20 (8.6)	26 (19.3)	67 (12.6)	
**Persons living/house**					0.610
up to 5	73 (44.2)	93 (40.1)	61 (44.5)	227 (42.5)	
6+	92 (55.8)	139 (59.9)	76 (55.5)	307 (57.5)	
**Persons sleeping/bedroom**					<0.001
1–3	109 (66.1)	125 (33.9)	48 (35.0)	282 (52.8)	
4+	56 (33.9)	107 (46.1)	89 (65.0)	252 (47.2)	
**Drinking water supply**					<0.001^1^
house tap water	69 (42.1)	120 (51.9)	39 (28.5)	228 (42.9)	
public tap water	93 (56.7)	104 (45.0)	98 (71.5)	295 (55.5)	
other	2 (1.2)	7 (3.0)	0 (0.0)	9 (1.7)	
**Electricity supply**					<0.001
public power corporation	157 (95.7)	163 (70.6)	105 (76.6)	425 (79.9)	
generator	0 (0.0)	36 (15.6)	15 (10.9)	51 (9.6)	
no electricity	7 (4.3)	32 (13.9)	17 (12.4)	56 (10.5)	
**Heating**					0.003
no	150 (90.9)	222 (95.7)	136 (99.3)	508 (95.1)	
yes	15 (9.1)	10 (4.3)	1 (0.7)	26 (4.9)	
**Toilet inside the house**					<0.001
no	42 (25.6)	74 (31.9)	87 (63.5)	203 (38.1)	
yes	122 (74.4)	158 (68.1)	50 (36.5)	330 (61.9)	
**Smoking pack years**					0.263
0	50 (30.0)	86 (37.0)	38 (28.0)	174 (33.0)	
1–30	81 (49.0)	95 (41.0)	70 (51.0)	246 (46.0)	
31+	32 (19.0)	49 (21.0)	27 (20.0)	108 (20.0)	
**Alcohol consumption**					0.112
yes	78 (47.3)	118 (50.9)	81 (59.1)	277 (51.9)	
no	87 (52.7)	114 (49.1)	56 (40.9)	257 (48.1)	
**Glasses of alcohol/last week**					0.001
0	99 (60.4)	148 (64.3)	98 (71.5)	345 (65.0)	
1–7	44 (26.8)	30 (13.0)	19 (13.9)	93 (17.5)	
8+	21 (12.8)	52 (22.6)	20 (14.6)	93 (17.5)	
**Days of alcohol/last week**					0.170
0	99 (60)	148 (64.1)	98 (71.5)	345 (64.7)	
1–2	51 (30.9)	60 (26.0)	25 (18.2)	136 (25.5)	
3+	15 (9.1)	23 (10.0)	14 (10.2)	52 (9.8)	

^1^ For the *p*-value estimation of this variable, category “other” was omitted; The DK/NA numbers were omitted as they were very low (0.2–0.6%).

**Table 2:** Self-perceived health status, the presence of chronic diseases, and anxiety and depression symptoms of Roma living in Roma settlements in Greece by gender.

	N (%)			*p*-Value ^2^
	Women	Men	Total	
**Self-perceived health status**				
very good or good	147 (51.2)	183 (74.1)	330 (61.9)	<0.001
fair	100 (34.8)	38 (15.4)	138 (25.9)	
very bad or bad	39 (13.6)	26 (10.5)	65 (12.2)	
**Presence of**				
at least one chronic disease	163 (58.4)	91 (37.9)	254 (48.9)	<0.001
one chronic disease	97 (35.4)	66 (27.8)	163 (31.9)	<0.001
>1 chronic disease	66 (23.0)	25 (10.1)	91 (17.0)	<0.001
high cholesterol	35 (13.7)	21 (9.6)	56 (11.8)	0.170
diabetes mellitus	26 (9.8)	15 (6.8)	41 (8.4)	0.237
high blood pressure	51 (18.9)	26 (11.8)	77 (15.7)	0.032
heart diseases	30 (11.1)	13 (5.7)	43 (8.7)	0.033
respiratory system diseases	31 (11.4)	15 (6.6)	46 (9.2)	0.061
tuberculosis	0 (0.0)	1 (0.4)	1 (0.2)	0.455 ^3^
cancer	3 (1.1)	1 (0.4)	4 (0.8)	0.402
anemia	25 (9.1)	7 (3.1)	32 (6.4)	0.005
depression	42 (15.9)	7 (3.1)	49 (10.1)	<0.001
other diseases				
other mental disease and drug abuse	5 (1.7)	10 (4.0)	15 (2.8)	
endocrine	18 (6.3)	0 (0.0)	18 (3.4)	
neurological	11 (4.0)	5 (2.0)	16 (3.0)	
musculoskeletal	7 (2.4)	4 (1.6)	11 (2.1)	
peptic	5 (1.7)	4 (1.6)	9 (1.7)	
communicable	2 (0.7)	2 (0.8)	4 (0.7)	
anxiety symptoms	136 (47.4)	84 (34.0)	220 (41.2)	0.002
depression symptoms	125 (43.6)	71 (28.7)	196 (36.7)	<0.001

^2^ For the *p*-value estimation in this Table, the Pearson Chi-Square test was used, unless stated otherwise; ^3^ for the *p*-value estimation of this variable Fisher’s Exact Test was used.

**Table 3:** Factors associated with the self-perceived health status of Roma living in Roma settlements in Greece. Results from multivariable logistic regression comparing those who had good or very good self-perceived health status with those who had fair, bad, or very bad self-perceived health status.

	Good/Very Good Self-Perceived Health Status	
	Adjusted OR (95% CI)	*p*-Value
**Prefecture**		
Attica ^†^	1	
Cen. Macedonia	1.16 (0.60 to 2.22)	0.663
Peloponnese	1.93 (0.95 to 3.91)	0.069
Thessaly	3.80 (1.92 to 7.52)	<0.001
**Settlement type**		
shacks ^†^	1	
houses	0.46 (0.25 to 0.86)	0.014
mixed	0.76 (0.42 to 1.36)	0.352
**Gender**		
men ^†^	1	
women	0.51 (0.32 to 0.82)	0.005
**Age group**		
18–29 ^†^	1	
30–45	0.44 (0.25 to 0.77)	0.004
>45	0.10 (0.05 to 0.19)	<0.001
**Living with a partner**		
no ^†^	1	
yes	2.14 (1.23 to 3.73)	0.007
**Presence of depression symptoms**		
no ^†^	1	
yes	0.18 (0.11 to 0.29)	<0.001
**Food insecurity**		
Never ^†^	1	
rare/sometimes/very often	0.51 (0.31 to 0.84)	0.008
**Glasses of alcohol/last week**		
0 ^†^	1	
1–7	2.24 (1.16 to 4.30)	0.016
8+	0.90 (0.48 to 1.71)	0.749

^†^ Reference category.

**Table 4:** Factors associated with the odds of the presence of a chronic disease of Roma living in Roma settlements in Greece: Results from multivariable logistic regression.

	Presence of a Chronic Disease	
	Adjusted OR (95% CI)	*p*-Value
**Settlement type**		
shacks ^†^	1	
houses	1.77 (1.04 to 3.00)	0.034
mixed	0.85 (0.51 to 1.42)	0.553
**Gender**		
men^†^	1	
women	1.70 (1.14 to 2.53)	0.009
**Age group**		
18–29 ^†^	1	
30–45	1.91 (1.18 to 3.09)	0.008
>45	8.41 (4.92 to 14.03)	<0.001
**Presence of anxiety symptoms**		
no ^†^	1	
yes	1.82 (1.20 to 2.77)	0.004
**Food insecurity**		
never ^†^	1	
rare/sometimes/very often	1.62 (1.05 to 2.48)	0.027
**Persons living in the house**		
6+ ^†^	1	
up to 5	1.62 (1.06 to 2.47)	0.025

^†^ Reference category.

## Data Availability

Data will be available upon a reasonable request addressed to the Hprolipsis project steering committee’ chair Giota Touloumi, email: gtouloum@med.uoa.gr.

## Appendix A

**Table A1:** The 4-item Patient Health Questionnaire (PHQ-4).

Over the Past 2 Weeks Have You Been Bothered by These Problems?	Not at All	Several Days	More Days Than Not	Nearly Every Day
Feeling down, depressed, or hopeless *	0	1	2	3
Little interest or pleasure in doing things *	0	1	2	3
Feeling nervous, anxious, or on edge **	0	1	2	3
Not being able to stop or control worrying **	0	1	3	3

* Items for depression scale, ** Items for anxiety scale.

**Table A2:** The 3-item household food insecurity questionnaire.

During the Last Month:	Never	Rare or Sometimes	Often	I Would Rather Not Answer
Was there ever no food at all in your household because there were no resources to get more?	0	1	2	
Did you or any household member go to sleep at night hungry because there was not enough food?	0	1	2	
Did you or any household member go a whole day without eating anything because there was not enough food?	0	1	2	

## References

[1] Kóczé A., Rövid M., Kaldor M., Moore H.L., Selchow S., Murray-Leach T. (2012). Pro-Roma Global Civil Society: Acting for, with or Instead of Roma?. Global Civil Society 2012: Ten Years of Critical Reflection.

[2] Marushiakova-Popova E., Popov V. (2018). Roma Labelling: Policy and Academia. Slovensky Narodopis.

[3] Council of Europe (CoE) (2012). Council of Europe Descriptive Glossary of Terms Relating to Roma Issues.

[4] European Union Agency for Fundamental Rights (FRA) (2019). Fundamental Rights Report 2019.

[5] Council of Europe (CoE) 1.0 From India to Europe, Fact Sheets on Roma History, Project Education of Roma Children in Europe. https://rm.coe.int/from-india-to-europe-factsheets-on-romani-history/16808b18ed.

[6] Council of Europe (CoE) 2.0 Arrival in Europe, Fact Sheets on Roma History, Project Education of Roma Children in Europe. http://rm.coe.int/arrival-in-europe-factsheets-on-romani-history/16808b1908.

[7] Marushiakova E., Popov V., Kamusella T., Jaskułowski K. (2009). Gypsy Slavery in Wallachia and Moldavia. Nationalisms Today.

[8] Council of Europe (CoE) 2.4 Western Europe, Fact Sheets on Roma History, Project Education of Roma Children in Europe. https://rm.coe.int/western-europe-factsheets-on-romani-history/16808b19e4.

[9] Council of Europe (CoE) 4.1 Austria and Hungary 1850–1938, Fact Sheets on Roma History, Project Education of Roma Children in Europe. https://rm.coe.int/austria-and-hungary-1850-1938-factsheets-on-romani-history/16808b1a93.

[10] Council of Europe (CoE) 5.0 Holocaust, Fact Sheets on Roma History, Project Education of Roma Children in Europe. http://rm.coe.int/holocaust-factsheets-on-romani-history/16808b1ab0.

[11] Cherkezova S., Tomova I. (2013). An Option of Last Resort? Migration of Roma and Non-Roma from CEE Countries.

[12] Albert G., Szilvasi M. (2017). Intersectional Discrimination of Romani Women Forcibly Sterilized in the Former Czechoslovakia and Czech Republic. Health Hum. Rights.

[13] Zampas C., Lamačková A. (2011). Forced and Coerced Sterilization of Women in Europe. Int. J. Gynecol. Obstet..

[14] Korver R. (2020). Framework for National Roma Integration Strategies up to 2020: European Implementation Assessment.

[15] Fresno J.M., Lajčáková J., Szira J., Mačáková S., Karoly M., Rossi M. (2019). A Meta-Evaluation of Interventions for Roma Inclusion.

[16] Frazer H., Marlier E. (2011). Promoting the Social Inclusion of Roma: Synthesis Report.

[17] Kagin J., Ivanov A. (2014). Roma Poverty from a Human Development Perspective.

[18] Parekh N., Rose T. (2011). Health Inequalities of the Roma in Europe: A Literature Review. Cent. Eur. J. Public Health.

[19] Zeman C.L., Depken D.E., Senchina D.S. (2003). Roma Health Issues: A Review of the Literature and Discussion. Ethn. Health.

[20] Cook B., Wayne G.F., Valentine A., Lessios A., Yeh E. (2013). Revisiting the Evidence on Health and Health Care Disparities among the Roma: A Systematic Review 2003–2012. Int. J. Public Health.

[21] Hassler S., Eklund L. (2012). Sense of Coherence and Self-Reported Health among Roma People in Sweden—A Pilot Study. Int. J. Circumpolar. Health.

[22] Parry G., Van Cleemput P., Peters J., Walters S., Thomas K., Cooper C. (2007). Health Status of Gypsies and Travellers in England. J. Epidemiol. Community Health.

[23] UCL Institute of Health Equity (2014). Review of Social Determinants and the Health Divide in the WHO European Region: Final Report.

[24] Solar O., Irwin A. (2010). A Conceptual Framework for Action on the Social Determinants of Health. Social Determinants of Health Discussion Paper 2 (Policy and Practice).

[25] World Health Organization (WHO) (2008). Closing the Gap in a Generation: Health Equity through Action on the Social Determinants of Health.

[26] Hajioff S., McKee M. (2000). The Health of the Roma People: A Review of the Published Literature. J. Epidemiol. Community Health.

[27] Monasta L., Erenbourg A., Restaino S., Lutje V., Ronfani L. (2012). Review of the Scientific Literature on the Health of the Roma and Sinti in Italy. Ethn. Dis..

[28] Koupilová I., Epstein H., Holčík J., Hajioff S., McKee M. (2001). Health Needs of the Roma Population in the Czech and Slovak Republics. Soc. Sci. Med..

[29] Wallengren S., Mellgren C. (2015). The Other Way Around: The Roma Minority’s View on Doing Research on Sensitive Topics. Int. J. Soc. Sci. Stud..

[30] Messing V. (2014). Methodological Puzzles of Surveying Roma/Gypsy Populations. Ethnicities.

[31] Code of Greek Nationality. https://www.legislationline.org/documents/id/5394.

[32] Bulbul G. (2017). Civil Society Monitoring Report on Implementation of the National Roma Integration Strategies in Greece: Focusing on Structural and Horizontal Preconditions for Successful Implementation of the Strategy.

[33] European Union Agency for Fundamental Rights (FRA) (2017). Second European Union Minorities and Discrimination Survey Technical Report.

[34] European Union Agency for Fundamental Rights (FRA) (2013). Roma Pilot Survey Technical Report: Methodology, Sampling and Fieldwork.

[35] Ministry of Labor and Social Insurance (MLSI) (2000). Rom Network Panhellenic Census Study Investigating the Social, Housing Conditions and Needs of Greek Gypsy Citizens [Πανελλήνια Aπογραφική Μελέτη Διερεύνησης Κοινωνικών, Oικιστικών Συνθηκών Και Aναγκών Των Ελλήνων Τσιγγάνων Πολιτών].

[36] Fundación Secretariado Gitano (FSG) Health and the Roma Community, Analysis of the Situation in Europe Bulgaria, Czech Republic, Greece, Portugal, Romania, Slovakia, Spain; No 97. http://www.gitanos.org/upload/78/83/Health_and_the_Roma_Community.pdf.

[37] Ministry of Employment and Social Protection, General Secretariat for the Management of Community and Other Resources (MESP) (2008). Study for the Recording of the Current Situation of the Roma in Greece, Report of Actions and Preparation of an Action Plan for the 4th Programming Period [Εκπόνηση Μελέτης Για Την Καταγραφή Της Υφιστάμενης Κατάστασης Των ΡOΜA Στην Ελλάδα, Aπολογισμός Δράσεων Και Εκπόνηση Σχεδίου Δράσης Για Την 4η Προγραμματική Περίοδο].

[38] Pappa E., Chatzikonstantinidou S., Chalkiopoulos G., Papadopoulos A., Niakas D. (2015). Health-Related Quality of Life of the Roma in Greece: The Role of Socio-Economic Characteristics and Housing Conditions. Int. J. Environ. Res. Public Health.

[39] Touloumi G., Karakosta A., Sypsa V., Petraki I., Anagnostou O., Terzidis A., Voudouri N.M., Gavana M., Vantarakis A., Rachiotis G. (2020). Design and Development of a Viral Hepatitis and HIV Infection Screening Program (Hprolipsis) for the General, Greek Roma, and Migrant Populations of Greece: Protocol for Three Cross-Sectional Health Examination Surveys. JMIR Res. Protoc..

[40] Tolonen H., HES Manual (2016). Part B: Fieldwork Procedures.

[41] Touloumi G., Karakatsani A., Karakosta A., Sofianopoulou E., Koustenis P., Gavana M., Alamanos Y., Kantzanou M., Konstantakopoulos G., Chryssochoou X. (2019). National Survey of Morbidity and Risk Factors (EMENO): Protocol for a Health Examination Survey Representative of the Adult Greek Population. JMIR Res. Protoc..

[42] Kroenke K., Spitzer R.L., Williams J.B.W., Löwe B. (2009). An Ultra-Brief Screening Scale for Anxiety and Depression: The PHQ–4. Psychosomatics.

[43] Swindale A., Bilinsky P. (2006). Development of a Universally Applicable Household Food Insecurity Measurement Tool: Process, Current Status, and Outstanding Issues. J. Nutr..

[44] Ballard T., Coates J., Swindale A., Deitchler M. (2011). Household Hunger Scale: Indicator Definition and Measurement Guide.

[45] Organisation for Economic Co-Operation and Development (2017). OECD Health at a Glance 2017: OECD Indicators.

[46] Eurostat Share of Persons Aged 16 and Over with or without Long-Standing (Chronic) Health Problems, 2014 (%) Health 2015—Statistics Explained. https://ec.europa.eu/eurostat/statistics-explained/index.php?title=File:Share_of_persons_aged_16_and_over_with_or_without_long-standing_(chronic)_health_problems,_2014_(%25)_Health2015.png.

[47] European Union Agency for Fundamental Rights (FRA) Survey on Minorities and Discrimination in EU (2016). https://fra.europa.eu/en/publications-and-resources/data-and-maps/survey-data-explorer-second-eu-minorities-discrimination-survey.

[48] European Union Agency for Fundamental Rights (FRA) Survey on Discrimination and Social Exclusion of Roma in EU. https://fra.europa.eu/en/publications-and-resources/data-and-maps/survey-discrimination-and-social-exclusion-roma-eu-2011.

[49] Belak A., Madarasova Geckova A., van Dijk J.P., Reijneveld S.A. (2017). Health-Endangering Everyday Settings and Practices in a Rural Segregated Roma Settlement in Slovakia: A Descriptive Summary from an Exploratory Longitudinal Case Study. BMC Public Health.

[50] Eklund Karlsson L., Ringsberg K.C., Crondahl K. (2017). Work-Integrated Learning and Health Literacy as Catalysts for Roma Empowerment and Social Inclusion: A Participatory Action Research. Action Res..

[51] Dotcho M. (2012). The Health Situation of Roma Communities: Analysis of the Data from the UNDP/World Bank/EC Regional Roma Survey 2011.

[52] Antonovsky A. (1979). Health, Stress and Coping. New Perspectives on Mental and Physical Well-Being.

[53] Janevic T., Jankovic J., Bradley E. (2012). Socioeconomic Position, Gender, and Inequalities in Self-Rated Health between Roma and Non-Roma in Serbia. Int. J. Public Health.

[54] Aisa R., Larramona G., Pueyo F. (2016). Discrimination and Self-Reported Health for the Spanish Roma. Public Health.

[55] Publications Office of the European Union The 10 Common Basic Principles on Roma Inclusion: Vademecum. https://op.europa.eu:443/en/publication-detail/-/publication/7573706d-e7c4-4ece-ae59-2b361246a7b0.

[56] Sordo Ruz T. (2019). Guide on Intersectional Discrimination. The Case of Roma Women.

[57] World Health Organization (WHO) (2019). Evidence and Resources to Act on Health Inequities, Social Determinants and Meet the SDGs.

[58] Robayo M., Millan N. (2019). Breaking the Cycle of Roma Exclusion in the Western Balkans.

[59] Gecková A.M., Babinská I., Bobáková D., Veselská Z.D., Bosáková L., Kolarcik P., Jarcuska P., Pella D., Halánová M., HepaMeta Team (2014). Socioeconomic Characteristics of the Population Living in Roma Settlements and Their Association with Health and Health-Related Behaviour. Cent. Eur. J. Public Health.

[60] Masseria C., Mladovsky P., Hernández-Quevedo C. (2010). The Socio-Economic Determinants of the Health Status of Roma in Comparison with Non-Roma in Bulgaria, Hungary and Romania. Eur. J. Public Health.

[61] European Union Agency for Fundamental Rights (FRA) (2016). Second European Union Minorities and Discrimination Survey: Roma—Selected Findings.

[62] Petraki I. (2014). Educational Program Young Roma Health Mediators. A Practical Guide for Implementing Health Promotion Activities with the Roma People.

[63] Eriksson M., Lindström B. (2006). Antonovsky’s Sense of Coherence Scale and the Relation with Health: A Systematic Review. J. Epidemiol. Community Health.

